# Selective Oxidation of Veratryl Alcohol over Au-Pd/Ce_0.62_Zr_0.38_O_2_ Catalysts Synthesized by Sol-Immobilization: Effect of Au:Pd Molar Ratio

**DOI:** 10.3390/nano8090669

**Published:** 2018-08-28

**Authors:** Carol M. Olmos, Lidia E. Chinchilla, Andrea M. Cappella, Alberto Villa, Juan J. Delgado, Ana B. Hungría, Ginesa Blanco, Jose J. Calvino, Laura Prati, Xiaowei Chen

**Affiliations:** 1 Departamento de Ciencia de los Materiales, Ingeniería Metalúrgica y Química Inorgánica, Facultad de Ciencias, Universidad de Cádiz, Campus Río San Pedro, E-11510 Puerto Real (Cádiz), Spain; carolmaritza1@gmail.com (C.M.O.); lidia.chinchilla@uca.es (L.E.C.); juanjose.delgado@uca.es (J.J.D.); ana.hungria@uca.es (A.B.H.); ginesa.blanco@uca.es (G.B.); jose.calvino@uca.es (J.J.C.); 2 Dipartimento di Chimica, Università degli Studi di Milano, I-20133 Milano, Italy; andreamarco.cappella@unimi.it (A.M.C.); alberto.villa@unimi.it (A.V.); laura.prati@unimi.it (L.P.)

**Keywords:** veratryl alcohol oxidation, lignin, bimetallic Au-Pd catalyst, ceria-zirconia, Au:Pd molar ratio

## Abstract

The selective oxidation of veratryl alcohol (VA), a model compound of lignin, with oxygen molecules to produce veratraldehyde (VAld) was studied over monometallic Au, Pd, and bimetallic Au:Pd nanoparticles supported on a Ce_0.62_Zr_0.38_O_2_ mixed oxide for the first time. These bimetallic Au-Pd catalysts with Au:Pd molar ratios from 0.4 to 4.3 were synthesized by the sol-immobilization method. Furthermore, all the catalysts were characterized by inductively coupled plasma-atomic emission spectroscopy (ICP-AES), N_2_ physisorption, X-ray photoelectron spectroscopy (XPS), scanning transmission electron microscopy-high angle annular dark field (STEM-HAADF) imaging, energy dispersive X-ray spectroscopy (EDXS), and temperature programmed reduction (TPR) techniques. A synergistic effect between gold and palladium was observed over all the bimetallic catalysts in a wide range of studied Au:Pd ratios. Remarkably, the optimum Au:Pd ratio for this reaction was 1.4 with a turnover frequency of almost six times larger than for the monometallic gold and palladium catalysts. Selectivity to veratraldehyde was higher than 99% for the monometallic Au, Pd, and all the bimetallic Au-Pd catalysts, and stayed constant during the reaction time.

## 1. Introduction

The development of strategies for the valorization of lignin to high-value chemicals is becoming increasingly important due to their potential application as sustainable supplements or replacements of fossil-based chemicals or fuels. Lignin is the second most abundant resource in nature after cellulose and accounts for about 25% of the world’s biomass. In addition, it is estimated that the pulp and paper industry produce around 70 million tons of lignin per year, which is burnt for heat and power generation [1]. Lignin is a three-dimensional polymer with methoxylated phenylpropane structures. Three primary monomers of lignin are p-coumaryl, coniferyl, and sinapyl alcohol and are combined with C-O (e.g., β-O-4, α-O-4, and 4-O-5) and C-C (e.g., β-5 and 5-5) bonds. The β-O-4 bond is the predominant linkage in lignin and occupies 50–60% of all the C-O linkages. It is highly desirable to open the lignin molecules to produce other chemicals, such as aromatics. Catalytic processes are promising strategies for the lignin transformations and can be divided into cracking (pyrolysis, fast thermolysis, hydrogenation, etc.), hydrolysis, hydrogenolysis, and reduction or oxidation reactions through homogeneous, heterogeneous, or enzymatic catalysis [2]. An attractive method for the valorization of lignin and lignin model compounds is oxidative depolymerization with environmentally-friendly oxidants, such as oxygen [3,4,5]. Since lignin contains many hydroxyl groups, it is susceptible to oxidation and oxidative depolymerization. The oxidative depolymerization/cracking cleaves the aryl ether bonds (for example, β-O-4 and 4-O-5) and other linkages within the lignin. The major oxidation products would be aromatic aldehydes or carboxylic acids, which depend on the reaction conditions [6]. Besides, because of the complexity of the structure of lignin feedstocks, the majority of academic research has focused on the oxidation of model compounds, such as 2-phenoxy-1-phenylethanol [7], vanillyl alcohol [8], 2-aryloxy-1-arylethanols [8], veratryl alcohol [4,9], apocynol [10], p-sinapyl alcohol [9], and p-hydroxy benzyl alcohol [9], among others. The studies on these model compounds could be relevant in terms of the reactivity of some motifs found in lignin, as well as how specific bonds (β-O-4, α-O-4, etc.) can be broken. In particular, the oxidation of veratryl alcohol to veratraldehyde is a benzylic oxidation representing the valorization of one of the β-O-4 model compounds of lignin [4,9].

Recently, heterogeneous catalysts have gained a lot of interest due to their excellent performance, stability, and reusability in selective oxidation under mild conditions, and especially in the oxidation of biomass-derived substrates [11]. In the literature, studies on heterogeneous catalysts for the oxidation of veratryl alcohol have focused mainly on monometallic catalysts [5], such as Co_3_O_4_ [9], Co-ZIF-9 [12], Mn/Al_2_O_3_, Co/Al_2_O_3_, Cu/Al_2_O_3_, Ag/Al_2_O_3_, Ru/Al_2_O_3_ and so on [13]. Bimetallic catalysts containing Au-Pd and Au-Pt have exhibited better catalytic performance and higher stability [14] than monometallic catalysts for the selective oxidation of alcohols [14,15,16]. Moreover, ceria is a reducible support and enhances the catalytic activities of Au-Pd nanoparticles for these reactions due to its easy exchange of Ce^3+^/Ce^4+^ [17,18]. Furthermore, the introduction of Zr^4+^ ions into the CeO_2_ fluorite lattice improves its oxygen storage capacity, the formation of oxygen vacancies [19], and the thermal stability with respect to the pure oxide [20]. To the best of our knowledge, no Au-Pd supported on ceria-zirconia mixed oxide catalyst has been reported for selective oxidation of veratryl alcohol up to now.

Based on the results in the literature, this work studied bimetallic Au-Pd supported on ceria-zirconia catalysts for the oxidation of veratryl alcohol. A series of monometallic Au and Pd and bimetallic Au-Pd supported on Ce_0.62_Zr_0.38_O_2_ catalysts have been synthesized using a sol-immobilization method. The total metal loadings were maintained at 1 wt %. The effect of Au:Pd molar ratio on the catalytic activity of oxidative transformation of veratryl alcohol has been investigated.

## 2. Materials and Methods

### 2.1. Catalyst Preparation

The support used in all the preparations was a Ce_0.62_Zr_0.38_O_2_ (CZ) mixed oxide kindly provided by Grace Davison (Maryland, MD, USA). The Brunauer-Emmett-Teller (BET)-specific surface area of this ceria-zirconia mixed oxide was 67 m^2^ g^−1^. 

The monometallic and bimetallic catalysts were synthesized by the sol-immobilization method as described in our previous work [21]. The nominal loadings of all the catalysts were 1 wt %. The Pd and Au precursors were Na_2_PdCl_4_ and NaAuCl_4_·2H_2_O, respectively. Pd and Au sols were prepared with their precursors and polyvinylpyrrolidone (PVP) and reduced by NaBH_4_ separately. The colloids of Au or Pd (acidified to pH = 2 by addition of sulfuric acid) were immobilized by adding 2 g of Ce_0.62_Zr_0.38_O_2_ mixed oxide support with vigorous stirring for 1 h. Then, the slurry was further filtered, washed with distilled water, and dried at 80 °C for 2 h. An oxidation treatment of the dried catalysts was performed in a flow of O_2_ at 250 °C for 1 h and purged with a flow of N_2_ during 1 h at the same temperature. The monometallic catalysts were labeled according to their Pd and Au loadings based on weight as determined from inductively coupled plasma-atomic emission spectroscopy (ICP-AES) analysis. The catalysts were 0.82%PdCZ and 0.86%AuCZ, in which 0.82% and 0.86% stand for the actual metal loadings. 

The first step of the bimetallic Au-Pd supported on ceria-zirconia catalyst preparation was the synthesis of Au sol. Firstly, 2.19 mL of NaAuCl_4_·2H_2_O (10 mg Au mL^−1^) solution and 2.19 mL PVP solution (1%, *w*/*w*) were added to 219 mL of H_2_O. After stirring for 2 min, 0.1 M NaBH_4_ (4.38 mL) was added under vigorous magnetic stirring. The ruby red Au sol immediately formed. After 3 min of sol generation, 3 g of Ce_0.62_Zr_0.38_O_2_ support was added to the Au sol under vigorous stirring. After 1 h of stirring, the slurry was filtered and washed with distilled water and dried for 15 min at 80 °C. The second step of the preparation was the deposition of Pd on the Au/Ce_0.62_Zr_0.38_O_2_ sample. For this purpose, the monometallic Au sample previously prepared was suspended in 250 mL of water at room temperature. H_2_ with a flow rate of 50 mL min^−1^ was bubbled into the suspension under atmospheric pressure and room temperature. Then, a mixture of 0.81 mL of Na_2_PdCl_4_ (10 mg Pd mL^−1^) solution and 0.81 mL of PVP solution (1%, *w*/*w*) was added to the suspension under magnetic stirring for 1 h. The resulting slurry was filtered and washed with distilled water and dried in air at 80 °C for 2 h. Subsequently, the dried catalyst was oxidized in a flow of O_2_ for 1 h at 250 °C and then was purged with a flow of nitrogen at the same temperature for 1 h. The catalyst was cooled to room temperature under the same flow and atmosphere. The obtained bimetallic catalyst was coded as 1.4AuPd-O where 1.4 is the actual Au:Pd molar ratio according to ICP results shown in Table 1. The concentrations of Au (III) and Pd (II) precursors have been varied to synthesize bimetallic catalysts with different Au:Pd molar ratios. The actual Au:Pd molar ratios of the final bimetallic catalysts were 0.4:1, 0.6:1, 1.4:1, 1.8:1, 3.7:1, and 4.3:1 (Table 1).

### 2.2. Catalyst Characterization

The gold and palladium loadings were determined by ICP-AES (Thermo Scientific, Waltham, MA, USA) from the diluted extract of aqua regia. A volumetric N_2_ adsorption at −196 °C using a Micromeritics ASAP-2020 instrument (Micromeritics, Norcross, GA, USA) was performed in order to determine BET-specific surface areas of the catalysts. XRD patterns of the catalysts were obtained using a Bruker diffractometer AXS (Bruker, Germany), Model D8 Advance, operated at 40 kV and 40 mA with Cu Kα radiation source (1.5418 Å). TPR experiments were carried out in a U-shaped quartz reactor filled with 100 mg of the catalyst. The samples were firstly pre-treated with 60 mL min^−1^ of helium for 30 min at room temperature. Then, a flow of 5% H_2_/Ar (60 mL min^−1^) was switched to the reactor. The temperature of the reactor was increased from room temperature to 900 °C with a heating rate of 10 °C min^−1^. A Pfeiffer Thermostar quadrupole mass spectrometer (Pfeiffer, Germany) was used to analyze the composition of the gases released from the outlet of the reactor.

The morphology, metal particle size distribution, and compositional information of the catalysts were studied using scanning transmission electron microscopy (STEM) on a JEOL2010 (JEOL, Tokyo, Japan) equipped with an Energy dispersive X-ray spectroscopy (XEDS) spectrometer Oxford INCA Energy 2000 system (Oxford instruments, Abingdon, UK). High angle annular dark field (HAADF)-STEM images were taken by an electron probe of 0.5 nm diameter at a diffraction camera length of 8 cm. More than 150 randomly selected metal particles were measured and corresponding metal particle size distributions were plotted. The average particle diameter (*d*) and total metal dispersion were calculated using a truncated cuboctahedron particle model [22] and homemade software (GAUSS). The STEM-XEDS technique provided compositional information for around 60 individual particles in each bimetallic catalyst.

XPS measurements were performed on a Kratos Axis Ultra DLD instrument (Kratos Analytical, Manchester, UK) with monochromatized Al Kα radiation (1486.6 eV). The spectrometer was operated in the constant analyzer energy mode. Pass energy of 160 eV was used for low resolution and wide range survey spectra, while 20 eV was used for high resolution and narrow core level spectra. The binding energy scale was calibrated with respect to the Zr 3d5/2 component of the mixed oxide support and fixed at 182.64 eV as reported in our previous work [23]. CasaXPS Software version 2.3.17dev6.3a, developed by Neal Fairley (Casa Software Ltd., UK), was employed for the XPS data analysis.

### 2.3. Catalytic Activity for Veratryl Alcohol Oxidation

Catalytic evaluation was carried out in a thermally controlled glass reactor of 30 mL, equipped with an electronically controlled magnetic stirrer connected to a large reservoir (5000 mL) containing oxygen [24]. A mass-flow controller was used to control the oxygen uptake. Veratryl alcohol and the catalyst (alcohol:total metal = 1000 mol:mol) were mixed in xylene (alcohol 0.3 M in xylene; total volume: 10 mL). The reactor was filled with 200 kPa of oxygen and then the reactor was heated to 80 °C under stirring. Periodic removal of samples from the reactor was performed. Identification and quantification of the products were done by comparison with the external standard calibrated samples by a gas chromatograph (HP 7820A) equipped with a capillary column (HP-5, 30 m, 0.32 mm, 0.25 μm Film, made by Agilent Technologies, Santa Clara, CA, USA) and a thermal conductivity detector.

## 3. Results and Discussion

### 3.1. Textural and Structural Properties

Table 1 lists the BET-specific surface areas and elemental analysis results of the monometallic and bimetallic catalysts. The BET-specific surface areas of the catalysts were very close to that of the Ce_0.62_Zr_0.38_O_2_ support. Furthermore, it can be observed that the actual Au and Pd loadings were lower than the expected values, indicating that the sol-immobilization method did not lead to complete deposition of Au and Pd on the Ce_0.62_Zr_0.38_O_2_ support. The Au and Pd losses during the synthesis were due to the weak bond between the Au and Pd sols and the support. Hence, part of the Au and Pd sols was removed during the filtering and washing steps [21].

**Figure 1:**
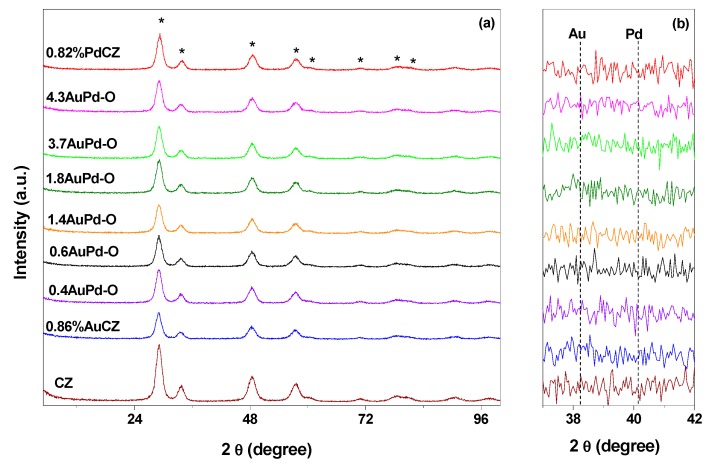
(**a**) XRD patterns of monometallic 0.86%AuCZ, 0.82%PdCZ and bimetallic Au-Pd catalysts with different Au:Pd molar ratios; (**b**) The enlarged diffraction profiles in the 2θ range between 37° and 42°. * Diffraction of the cubic fluorite structure for Ce_0.62_Zr_0.38_O_2_ mixed oxide.

The XRD patterns (Figure 1) of bimetallic and monometallic catalysts showed diffraction peaks corresponding to the crystallographic planes of the fluorite-type cubic structure of ceria-zirconia mixed oxide [20,25,26]. The metals were well dispersed on the ceria-zirconia support because no metallic palladium and/or gold, PdO or Au-Pd, alloy diffraction peaks were observed (Figure 1b) [16,21].

### 3.2. STEM Results

Representative STEM-HAADF images of the monometallic catalysts have been presented in our previous work [21]. Table 1 shows the average particle size and metal dispersion of these two catalysts. The 0.86%AuCZ catalyst possessed a wider particle size distribution than the 0.82%PdCZ catalyst: from 1 to 12 nm [21]. The 0.82%PdCZ catalyst presented a narrow particle size distribution and most of the particle sizes ranged from 0.5 to 5 nm. The average particle size of the 0.82%PdCZ and 0.86%AuCZ catalysts were 2.7 and 6.0 nm, respectively. Moreover, the metal dispersion of the 0.82%PdCZ and 0.86%AuCZ catalysts were 37% and 20%.

**Figure 2:**
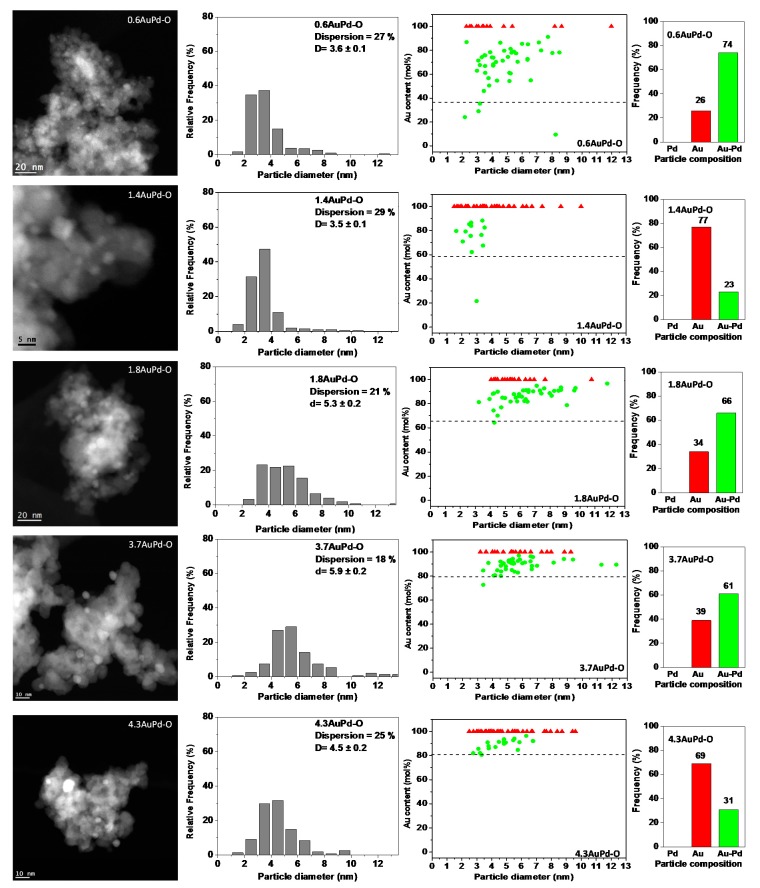
Scanning transmission electron microscopy-high angle annular dark field (STEM-HAADF) images, particle size distribution, relationship between the particle composition and size, and frequencies of Au, Pd, and Au-Pd particles of bimetallic catalysts determined by energy dispersive X-ray spectroscopy (EDXS). The dashed line is the actual Au composition obtained by inductively coupled plasma (ICP) analysis.

Figure 2 includes four types of graphs of each bimetallic catalyst: (1) representative STEM-HAADF image of the catalyst; (2) particle size distribution of the catalyst including monometallic Pd and Au, and bimetallic Au-Pd particles; (3) the composition of each analyzed nanoparticle obtained by the XEDS technique as a function of the size; and (4) relative frequency of each type of particle. The metal particle size of the bimetallic catalysts fell in the 0.5–17 nm range as shown in the particle size distribution of the catalysts. The average metal particle size and metal dispersion of the different bimetallic catalysts showed significant differences. The bimetallic 0.6AuPd-O and 1.4AuPd-O catalysts showed a similar average particle size (3.6 ± 0.1 nm and 3.5 ± 0.1 nm) with similar metal dispersion (27% and 29%). The 0.4AuPd-O, 1.8AuPd-O, 3.7AuPd-O, and 4.3AuPd-O catalysts presented larger average particle sizes (between 4.5 ± 0.2 nm and 5.9 ± 0.2 nm) and metal dispersion from 18% to 25%.

STEM-XEDS analyses were performed to obtain compositional information of the bimetallic catalysts, which was considered important based on catalytic activity data in Section 3.5. Around 60 individual particles were analyzed for each sample. In the figure of correlation between the content of Au versus the particle size, the dashed line indicates the Au content of each catalyst determined by ICP-AES (Figure 2). Monometallic Au and bimetallic Au-Pd nanoparticles were observed simultaneously by the STEM-XEDS technique. The Au content of the bimetallic particles was in the 10–96 mol% range. The composition-size diagrams indicate that 1.4AuPd-O and 4.3AuPd-O catalysts predominated the gold monometallic particles, while the 0.6AuPd-O, 1.8AuPd-O, and 3.7AuPd-O catalysts predominated the Au-Pd bimetallic particles. No Pd monometallic particles were detected. The low contrast between palladium and the heavy support (compared to gold) makes it more difficult to detect Pd when working in STEM mode, as shown in our previous work [16,21,25]. A recent paper from Hutchings´ group also stated this difficulty in observing Pd and Pd-containing nanoparticles on a ceria-zirconia mixed oxide support [27]. This fact could contribute to underestimating the fraction of palladium-rich nanoparticles (including monometallic palladium). In addition, no correlation was found between the Au and Pd content and the formation of bimetallic particles. On the other hand, an estimation of the average Au:Pd ratio measured by STEM-XEDS was calculated from the size and composition of the individual nanoparticles (Table 2). All the values ranged from 3.8 to 57, which were much higher than those determined by ICP-AES. The differences could be due to the low contrast between palladium and the heavy support detected by the STEM technique [21]. 

### 3.3. XPS Results

**Figure 3:**
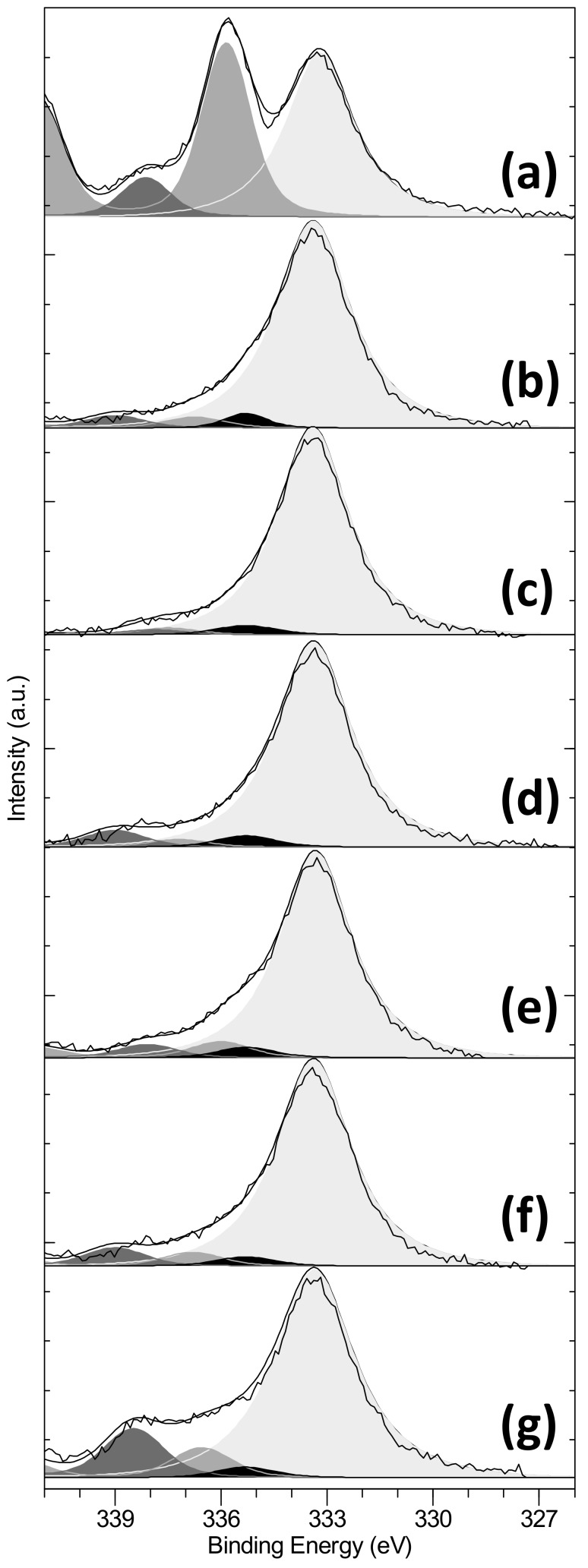
XPS high resolution spectra for samples listed in Table 3, corresponding to Zr 3p3/2 (light gray), Pd 3d5/2 (medium and dark gray corresponding to Pd^0^ and Pd^δ+^, respectively), and Au 4d5/2 (black): (**a**) 0.82%PdCZ; (**b**) 4.3AuPd-O; (**c**) 3.7AuPd-O; (**d**) 1.8AuPd-O; (**e**) 1.4AuPd-O; (**f**) 0.6AuPd-O; and (**g**) 0.4AuPd-O.

**Figure 4:**
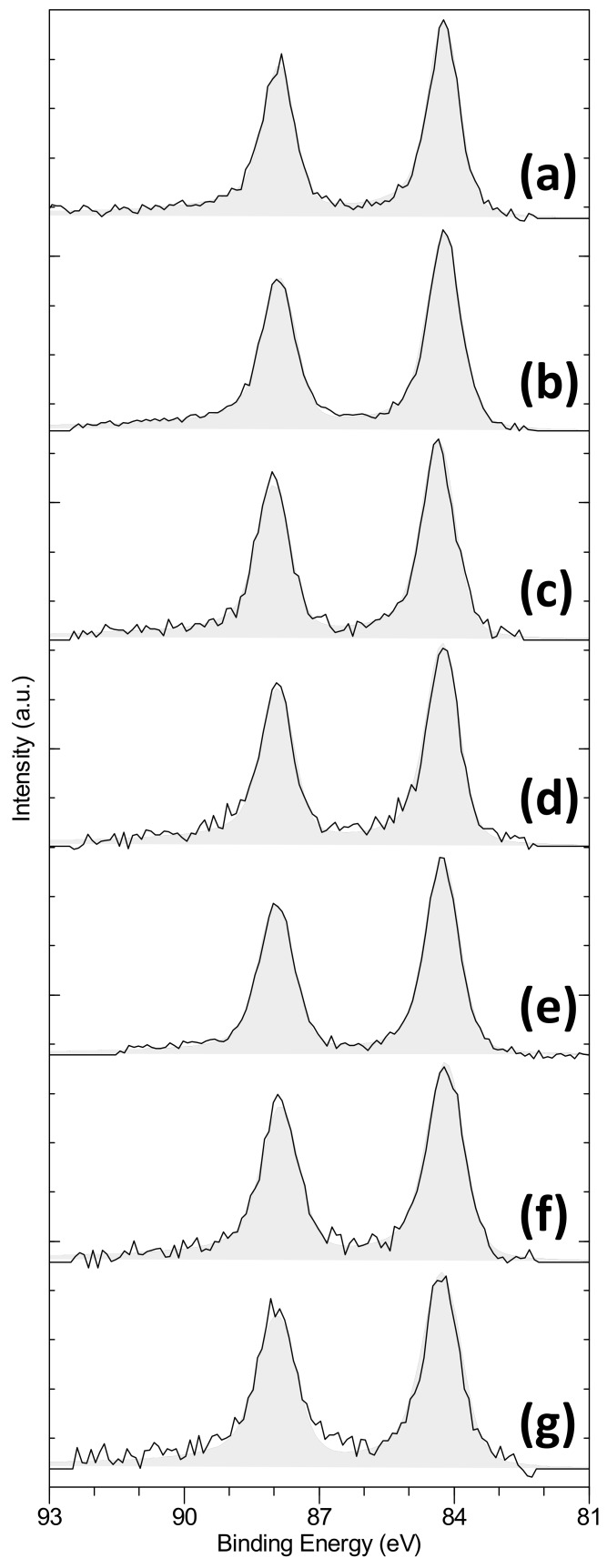
XPS high resolution spectra corresponding to Au 4f for samples listed in Table 3: (**a**) 0.86%AuCZ; (**b**) 4.3AuPd-O; (**c**) 3.7AuPd-O; (**d**) 1.8AuPd-O; (**e**) 1.4AuPd-O; (**f**) 0.6AuPd-O; and (**g**) 0.4AuPd-O.

The monometallic and bimetallic catalysts were characterized using the XPS technique to know the oxidation states and chemical composition on the surface of these catalysts. Figure 3 and Figure 4 show the Pd 3d and Au 4f X-ray photoelectron spectra of these catalysts. The Au 4f7/2 binding energy of about 84.1–84.4 eV corresponds either to a reduced phase of metallic Au^0^ (mostly like Au^0^) or larger Au particles [21,22,23,26]. Only metallic gold (Au^0^) was observed for the Au-containing catalysts. 

The XPS spectra of the Pd 3d region in the catalysts showed peaks of binding energy around 337.9 eV and 338.7 eV corresponding to the Pd^δ+^ species and metallic Pd^0^ species between 335.8 and 337.2 eV [21,28,29,30]. Table 3 lists the percentages of Pd^δ+^ and metallic Pd^0^ species of the catalysts. The 0.82%PdCZ catalyst showed 82% of Pd^0^, while the bimetallic catalysts presented lower percentages of Pd^0^ (between 39% and 56%). There were more oxidized Pd^δ+^ and less metallic Pd^0^ on the bimetallic catalysts compared with the monometallic 0.82%PdCZ catalyst. The differences in oxidation states of Pd on the monometallic 0.82%PdCZ catalyst and bimetallic catalysts can be attributed to Pd being easily oxidized on bimetallic catalysts with lower loadings of Pd than that of the monometallic 0.82%PdCZ catalyst. On the other hand, the STEM-HAADF results confirmed that all the Pd particles in the 0.82%PdCZ catalyst were smaller than 10 nm in diameter (Figure 2). Therefore, all the Pd atoms in this catalyst would have been analyzed by XPS since the sizes were in the XPS analysis range (radius was less than 5.0 nm) [16,21].

For the XPS data analysis, zirconium was considered as a reference for other elements on the catalysts due to its stability and homogeneous distribution in the mixed ceria-zirconia oxide support [21,25]. The Au:Zr and Pd:Zr molar ratios calculated with the XPS data reflect the gold and palladium available on the surface of the catalysts. Table 3 shows the Au:Zr, Pd:Zr, and Au:Pd molar ratios calculated by the XPS data. The Au:Zr molar ratio varied from 0.04 to 0.08. The Pd:Zr molar ratio decreased with the decrease in Pd content in the catalysts. The Au:Pd molar ratios calculated from the XPS results for 1.8AuPd-O, 3.7AuPd-O, and 4.3AuPd-O were 0.70, 1.41, and 2.20, which were lower than the ICP results, but much closer than those obtained by the XEDS technique. This result confirms that there were small Pd nanoparticles that could not be detected by STEM-HAADF. The 0.4AuPd-O, 0.6AuPd-O, and 1.4AuPd-O catalysts presented Au:Pd molar ratios close to the ICP results. Since XPS is a surface-sensitive technique, this result indicates that the bimetallic 0.4AuPd-O, 0.6AuPd-O, and 1.8AuPd-O catalysts had a “Pd-enriched surface” and the bimetallic 1.4AuPd-O, 3.7AuPd-O, and 4.3AuPd-O catalysts presented an “Au-enriched surface.”

The Ce^3+^ percentages in total cerium amount of all the catalysts were also calculated by XPS (Table 3), being in the range of 10 to 30%.

### 3.4. TPR Results

**Figure 5:**
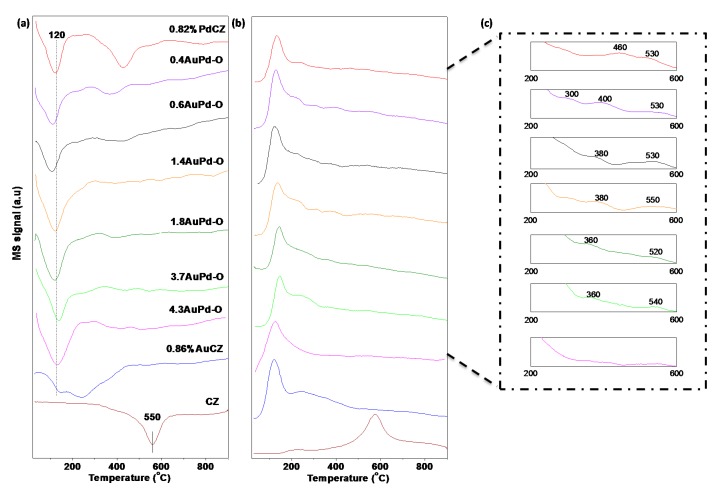
Temperature programmed reduction (TPR) profiles of the monometallic and bimetallic catalysts and support: (**a**) consumption of hydrogen; (**b**) formation of H_2_O; (**c**) the enlarged H_2_O formation profiles between 200 °C and 600 °C.

H_2_-temperature programmed reduction (H_2_-TPR) was performed to investigate the redox properties of the catalysts, as well as the interaction degree between metal and support. Figure 5 shows the TPR profiles of the catalysts and the support. The reduction peak at 550 °C of ceria-zirconia support can be assigned to the reduction of Ce^4+^. The TPR profile of the 0.86%AuCZ catalyst indicates that addition of Au to the support led to a much lower reduction temperature at 150 °C [31]. The profiles of the monometallic 0.82%PdCZ and bimetallic catalysts showed a first reduction peak around 120 °C, which can be attributed to the reduction of the support (Ce^4+^ → Ce^3+^) [32] and oxidized Pd^δ+^ species weakly interacting with the support [32,33]. 

The reduction temperature in all the bimetallic catalysts was closer to that of the monometallic Pd catalysts, suggesting that a small content of palladium, such as is the case of the 4.3AuPd-O catalyst, resulted in an enhancement of reducibility of catalyst. The 0.82%PdCZ, 0.4AuPd-O, 0.6AuPd-O, 1.4AuPd-O, 1.8AuPd-O, and 3.7AuPd-O catalysts also exhibited H_2_ consumption peaks in the range of 300–600 °C, which are associated with the reduction of Pd^δ+^ species that interact strongly with the CZ support [32]. For the 4.3AuPd-O catalyst, a reduction peak at high temperatures was not detected, possibly because of a low Pd loading on this catalyst (<0.1 wt %). The TPR profiles of Pd-containing catalysts obtained from the evolution of H_2_ consumption did not display a negative peak characteristic of the formation of Pd β-hybrid in the temperature range 50–100 °C. The absence of this peak indicates that PdO was highly dispersed, which is in good accordance with the XRD results [32].

The existence of Pd^δ+^ species on the surface of the Pd-containing catalysts has been proven by XPS results. Additionally, the addition of Au and Pd promoted reduction of the CZ support, which could be owing to the metal-CZ support interactions and the H_2_ spillover from metals to the CZ support. 

### 3.5. Catalytic Activity for Veratryl Alcohol Oxidation

**Figure 6:**
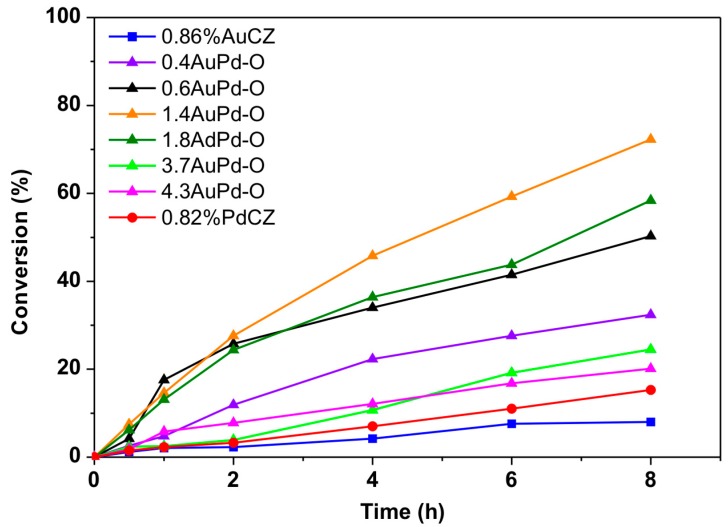
Catalytic activities of veratryl alcohol oxidation in the liquid phase as a function of time in the presence of the monometallic or bimetallic catalysts. Reaction conditions: alcohol:total metal ratio = 1000 mol:mol, alcohol 0.3 M, reaction temperature: 80 °C. The reactor was pressurized at 200 kPa of oxygen.

Veratryl alcohol conversion is presented as a function of time in Figure 6. In all cases, the monometallic and bimetallic catalysts showed an increase of conversion with reaction time. After 8 h of reaction, the monometallic 0.86%AuCZ catalyst exhibited the lowest veratryl alcohol conversion of 8% while the 0.82%PdCZ catalyst showed 15.3%. A synergistic effect can be observed on all the bimetallic Au-Pd catalysts evaluated, which exhibited higher catalytic activity than both monometallic catalysts. In addition, the 1.4AuPd-O catalyst with a conversion of 72.3% was the most active catalyst among the monometallic and bimetallic catalysts.

**Scheme 1:**
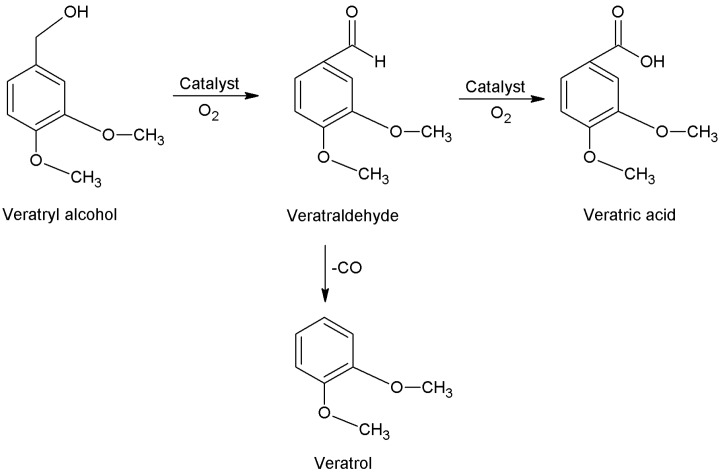
Reaction pathway of selective oxidation of veratryl alcohol.

The possible reaction pathway and products of selective oxidation of veratryl alcohol are shown in Scheme 1. The first step of oxidation of veratryl alcohol is to form veratraldehyde. There are two reaction routes for veratraldehyde. On the one hand, further oxidation of veratraldehyde can produce veratric acid. On the other hand, a carbonyl group can be eliminated to form veratrole. In this work, no other product except veratraldehyde was detected by gas chromatography-mass spectrometry. All the catalysts showed a selectivity >99% to veratraldehyde during the reaction time of 8 h. 

**Figure 7:**
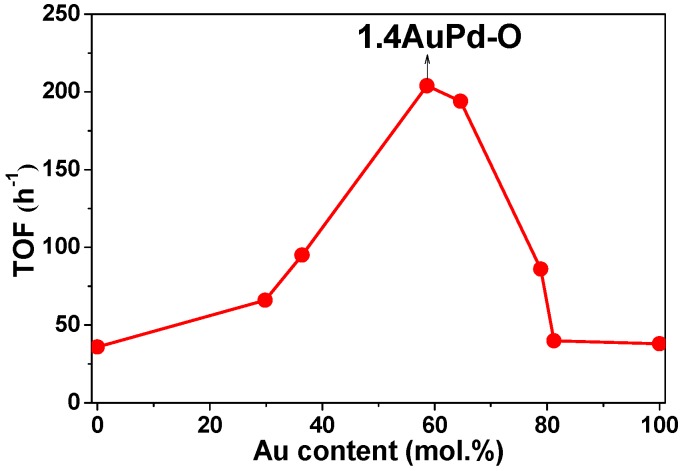
Turnover frequency (TOF) values over all the catalysts at reaction time of 0.5 h for selective oxidation of veratryl alcohol to produce veratraldehyde.

The initial turnover frequencies (TOFs) at a reaction time of 0.5 h shown in Table 1 and Figure 7 also confirm the enhanced catalytic activity of the 1.4AuPd-O catalyst with a factor of 5.7 times and 5.4 times compared with the 0.86%AuCZ and 0.82%PdCZ catalysts, respectively. The highest TOF was obtained when the Au:Pd ratio was 1.4. It can be observed that the 3.7AuPd-O and 4.3AuPd-O catalysts with relatively lower Pd content exhibited slightly more activity than the monometallic 0.86%AuCZ and 0.82%PdCZ catalysts. The order of the TOF values was 0.86%AuCZ < 0.82%PdCZ < 4.3AuPd-O < 0.4AuPd-O < 3.7AuPd-O < 0.6AuPd-O < 1.8AuPd-O < 1.4AuPd-O.

As shown in Table 1, all catalysts presented a similar surface area (~66 m^2^ g^−1^). With regard to oxidation state of the two metals, the XPS data analysis indicates the coexistence of metallic Pd^0^ and oxidized Pd^δ+^ species in all the catalysts, while gold was only present as metallic Au^0^. The catalyst with the highest conversion (1.4AuPd-O) presented similar metallic Pd^0^ and oxidized Pd^δ+^ species as the 4.3AuPd-O catalysts with lower conversion. The influence of the Pd oxidation states could not be established in this sense.

It is well known that the Au:Pd ratio can affect the catalytic behavior in oxidation reactions [16,26,27,34]. The bimetallic Au-Pd supported on ceria-zirconia catalysts with a wide Au:Pd ratio of 0.4–4.3 and total metal loading of 1 wt % showed a synergistic effect. The 1.4AuPd-O catalyst with an Au:Pd molar ratio of 1.4 and average particle size around 3.6 nm showed the best catalytic activity. The 1.8AuPd-O catalyst with the higher average particle size (5.3 nm) exhibited a slightly lower TOF value than the best catalyst 1.4AuPd-O, as shown in Table 1 and Figure 7. These results certify that the metal particle size is not the only determining parameter for catalytic activity. For this reason, it can be concluded that the Au:Pd molar ratio is one key factor that modulates the catalytic behavior for the oxidation of veratryl alcohol.

STEM results showed a considerable change in particle size and bimetallic percentage particles on the catalysts. Due to the difficulties in visualizing small Pd nanoparticles over a heavy ceria-zirconia support using the STEM-HAADF technique [14,19,24,26], the percentages of Au, Pd, and bimetallic Au-Pd particles could provide some information about the composition of the metal particles over these catalysts, but they are not very relevant. This result suggests that the composition of the particles play an important but not unique role on veratryl alcohol conversion to veratraldehyde. In addition, it is clear that the appearance of bimetallic particles increases to an increase in the conversion values, from 8% and 15.3% in 0.86%AuCZ and 0.82%PdCZ, respectively, to 20.1% in 4.3AuPd-O. Over all the monometallic and bimetallic catalysts studied in this work, the main product veratraldehyde was found with selectivity higher than 99%. This result indicates that the selectivity to veratraldehyde is independent of the coexistence of monometallic and bimetallic particles. This synergy effect observed between Au and Pd over ceria-zirconia mixed oxide support in the form of small nanoparticles provides interesting information. Finally, the results make evident the correlation between factors such as the Au:Pd molar ratio, the frequency of bimetallic particles and Pd content, and Pd oxidation states with the catalytic activity of selective oxidation of veratryl alcohol, which is very complicated and beyond the achievement of this work.

## 4. Conclusions

Bimetallic Au:Pd supported on ceria-zirconia mixed oxide catalysts prepared by the sol-immobilization method have been employed for the first time for the selective oxidation of veratryl alcohol to produce veratraldehyde. The influence of the Au:Pd molar ratios on the catalytic activities for this catalytic reaction has been investigated. The optimized Au:Pd molar ratio found was 1.4, with veratryl alcohol conversion of 72% and selectivity toward veratraldehyde of 99%. Factors such as the Au:Pd molar ratio, bimetallic particle content, and the co-existence of metallic Pd^0^ and oxidized Pd^δ+^ could improve the enhanced catalytic activity for veratryl alcohol oxidation.

## Figures and Tables

**Table 1:** Physicochemical properties of the monometallic and bimetallic catalysts.

Catalyst	Au Loading (wt %)		Pd Loading (wt %)		Theoretical Au:Pd Molar Ratio	Actual Au:Pd Molar Ratio ^b^	Au Content (mol%)	S_BET_ (m^2^ g^−1^)	Average Particle Size (nm) ^c^	Metal Dispersion (%) ^c^	Conversion (%) ^d^	TOF (h^−1^) ^e^
	Theoretical	Actual ^a^	Theoretical	Actual ^a^								
0.86%AuCZ	1.0	0.86	-	-	-	-	100	67	6.0 ± 0.2	20	8.0	36
0.4AuPd-O	0.50	0.37	0.5	0.47	0.5	0.4	29.8	63	5.2 ± 0.2	21	32.4	66
0.6AuPd-O	0.6	0.36	0.4	0.34	0.8	0.6	36.4	66	3.6 ± 0.1	27	50.3	95
1.4AuPd-O	0.73	0.55	0.27	0.21	1.5	1.4	58.0	66	3.5 ± 0.1	29	72.3	204
1.8AuPd-O	0.8	0.54	0.2	0.16	2.2	1.8	64.6	66	5.3 ± 0.2	21	58.4	194
3.7AuPd-O	0.9	0.69	0.1	0.10	4.9	3.7	78.9	65	5.9 ± 0.2	18	24.5	86
4.3AuPd-O	0.92	0.64	0.08	0.08	6.2	4.3	81.2	66	4.5 ± 0.2	25	20.1	40
0.82%PdCZ	-	-	1.0	0.82	-	-	0	66	2.7 ± 0.1	37	15.3	38

^a^ Weight percentage per gram of sample obtained by inductively coupled plasma-atomic emission spectroscopy (ICP-AES) analysis; ^b^ Molar ratio was calculated by weight percentage of metal as determined by ICP-AES; ^c^ The average particle size and metal dispersion were determined by scanning transmission electron microscopy (STEM) technique; ^d^ Conversion at 8 h of reaction; ^e^ Turnover frequency (TOF) at 0.5 h of reaction.

**Table 2:** Estimation of composition of the Au-Pd catalyst derived from Energy-dispersive X-ray spectroscopy (XEDS) analysis.

Catalyst	Number of Particles in Each Composition Category			Mean Particle Size (nm) ^a^			Au Content in Bimetallic Particles (%) ^c^	Interval of Au Content in the Bimetallic Particles (%)	Au Content in All Particles (%) ^d^	Au:Pd Molar Ratio ^d^
	Au	Pd	Au-Pd	Au	Pd ^b^	Au-Pd				
0.6AuPd-O	16	-	45	4.5	-	4.8	69	10–91	79	3.8
1.4AuPd-O	43	-	13	3.9	-	2.7	73	22–88	98	57
1.8AuPd-O	22	-	40	5.9	-	6.5	89	64–97	93	12.8
3.7AuPd-O	25	-	40	6.1	-	5.8	90	72–97	95	18
4.3AuPd-O	44	-	20	5.2	-	4.7	91	81–96	98	48

^a^ Average metal particle size as obtained from particles analyzed by XEDS; ^b^ Not determined; ^c^ Atomic percentage of gold content as determined by XEDS considering only bimetallic particles; ^d^ Calculate from the data as determined by XEDS with respect to all particles analyzed.

**Table 3:** Quantified XPS data of bimetallic catalysts with different Au:Pd ratios.

Catalyst	Ce^3+^ (%)	Au 4f7/2		Pd 3d5/2				Molar ratios			
		Au^0^ (%)	Binding Energy of Au^0^ (eV)	Pd^0^ (%)	Binding Energy of Pd^0^ (eV)	Pd^δ+^ (%)	Binding Energy of Pd^δ+^ (eV)	Au:Zr	Pd:Zr	Au:Pd	Au Content (mol%)
0.86%AuCZ	25	100	84.2	-	-	-	-	0.06	-	-	
0.4AuPd-O	10	100	84.3	39	336.6	61	338.5	0.04	0.15	0.30	23
0.6AuPd-O	26	100	84.2	50	336.6	50	338.4	0.06	0.06	0.80	44
1.4AuPd-O	21	100	84.2	55	336.0	45	338.1	0.08	0.06	1.49	60
1.8AuPd-O	10	100	84.2	41	337.2	59	338.7	0.04	0.07	0.70	41
3.7AuPd-O	13	100	84.3	48	337.0	52	338.7	0.05	0.04	1.41	59
4.3AuPd-O	30	100	84.2	56	335.8	44	337.9	0.08	0.04	2.20	69
0.82%PdCZ	14	-	-	82	335.9	18	338.1	-	0.42	-	
